# Amniotic Fluid Embolism in Donor Egg Twin Pregnancy: A Clinical Challenge in Critical Care

**DOI:** 10.7759/cureus.84557

**Published:** 2025-05-21

**Authors:** Ahmed Owies, Ahmed Attia, Harrie Toms John, Reena Gajjar, Madiha Abbas

**Affiliations:** 1 Intensive Care Unit, Epsom and St. Helier University Hospitals NHS Trust, London, GBR; 2 Critical Care Medicine, Epsom and St. Helier University Hospitals NHS Trust, London, GBR

**Keywords:** amniotic fluid embolism (afe), donor egg, donor oocyte pregnancy, immune-mediated reactions, obstetric emergencies, peripartum complications, postpartum cardiomyopathy

## Abstract

Amniotic fluid embolism (AFE) is a rare but life-threatening obstetric emergency marked by respiratory distress, severe hypotension, and coagulopathy with haemorrhage. This case involves a 45-year-old nulliparous woman with a dichorionic diamniotic twin pregnancy via in vitro fertilisation (IVF) with a donor egg. At 36 weeks' gestation, the patient developed sudden respiratory distress following the detection of persistent maternal tachycardia. Cardiotocography (CTG) revealed foetal distress, necessitating an emergency caesarean section, which resulted in the delivery of two live neonates. Her postpartum course was complicated by haemorrhage managed with a vaginal balloon and a second episode of respiratory distress with hypoxia. Admitted to the intensive care unit (ICU) for supportive care, she recovered well. Further investigations excluded pulmonary embolism and infection. Whilst peripartum cardiomyopathy (PPCM) was considered, the presence of massive haemorrhage and disseminated intravascular coagulation (DIC) strongly suggested AFE. This case highlights AFE’s rare occurrence in an IVF pregnancy and supports the emerging hypothesis that conception through donor eggs may pose a significant, under-recognised immunologic risk for AFE.

## Introduction

Amniotic fluid embolism (AFE) is a rare, potentially lethal obstetric emergency characterised by abrupt respiratory failure, haemodynamic collapse, and, in many instances, disseminated intravascular coagulation (DIC). Despite substantial progress in obstetric and intensive care practices, AFE remains one of the leading causes of maternal death in high-resource regions with incidence rates between 1.7 to 6.1 per 100,000 deliveries and maternal case fatality of up to 40% [1]. The pathogenesis of AFE is not fully understood. Current models suggest that systemic inflammation following the entry of amniotic fluid or foetal antigens into the maternal circulation triggers an anaphylactoid reaction, rather than a true embolism [2].

Several predetermined AFE risk factors have been established from large-scale population studies, including advanced maternal age, multiparity, caesarean delivery, placenta previa, placenta abruption, induction of labour, and multiple gestation [3]. Growing interest has been directed towards immunologic contributors, specifically in the cases of pregnancies conceived via assisted reproductive technologies (ART). Among them, donor egg conception is a distinct immunological case where the mother’s immune system encounters a totally allogenic foetus, lacking any human leucocyte antigen (HLA) similarities between the mother and embryo. This genetic inequality is quite high compared to that in autologous ART or spontaneous conception [4].

There is a growing body of literature suggesting that donor egg conception may predispose to an increased risk of obstetric complications with presumed immunological underpinnings, such as preeclampsia and placental dysfunction [5,6]. However, evidence of its contribution towards AFE is rare. The incompatibility of the immunologic nature of foetal and maternal tissues may lead to heightened inflammatory reactions during critical obstetric events like labour, delivery, or postpartum haemorrhage (PPH). Whilst the correlation between donor oocyte pregnancies and AFE has not been conclusively determined, multiple cases and cohort studies reveal a possible connection especially in the context of multiples and operative delivery [7].

We report a case of a 45-year-old woman who achieved dichorionic diamniotic twin pregnancy following in vitro fertilisation (IVF) using donor ovum. She had an eventful postpartum course, complicated by respiratory failure, coagulopathy, and cardiac dysfunction indicative of AFE. This case is consistent with the emerging hypothesis that conception through donor eggs may pose a significant, under-recognised immunologic risk for AFE.

Peripartum cardiomyopathy (PPCM) is one very important differential diagnosis in this setting, which is a rare form of heart failure that presents during late pregnancy/puerperium and is clinically diagnosed by a lowered left ventricular (LV) ejection fraction (EF) (<45%) during late pregnancy/puerperium when no other identified cause exists [8]. Patients with PPCM may present with dyspnoea, pulmonary oedema, and multiorgan dysfunction and coexist with or be unmasked by obstetric events such as haemorrhage and infections [8]. In this case, LV dysfunction and mitral regurgitation (MR) were initially indicative of PPCM. However, the sudden onset of respiratory compromise, DIC, and characteristic imaging findings of bilateral ground-glass opacities and pleural effusions are more likely to be explained by AFE [9]. Whilst PPCM can coexist with AFE, distinguishing the two requires careful assessment of clinical course and investigations. In this case, the sudden onset of respiratory distress immediately postpartum, presence of coagulopathy and DIC, massive haemorrhage, and bilateral ground-glass opacities on computed tomography pulmonary angiogram (CTPA) were more consistent with AFE. In contrast, PPCM typically presents subacutely with isolated cardiac dysfunction and without DIC.

Given the increasing prevalence of ART and donor egg conception, this case underscores the importance of recognising potential immunologic complications such as AFE. Further investigation is warranted to elucidate the pathophysiological links between donor oocyte pregnancies and severe maternal outcomes.

## Case presentation

A 45-year-old woman, gravida 1 para 0, with a history of attention-deficit hyperactivity disorder (ADHD), conceived through IVF using donor eggs, resulting in a dichorionic diamniotic twin pregnancy. This followed multiple failed IVF attempts. Her previous gynaecological history was unremarkable, with regular menstrual cycles. Her partner’s semen analysis was normal.

**Figure 1:**
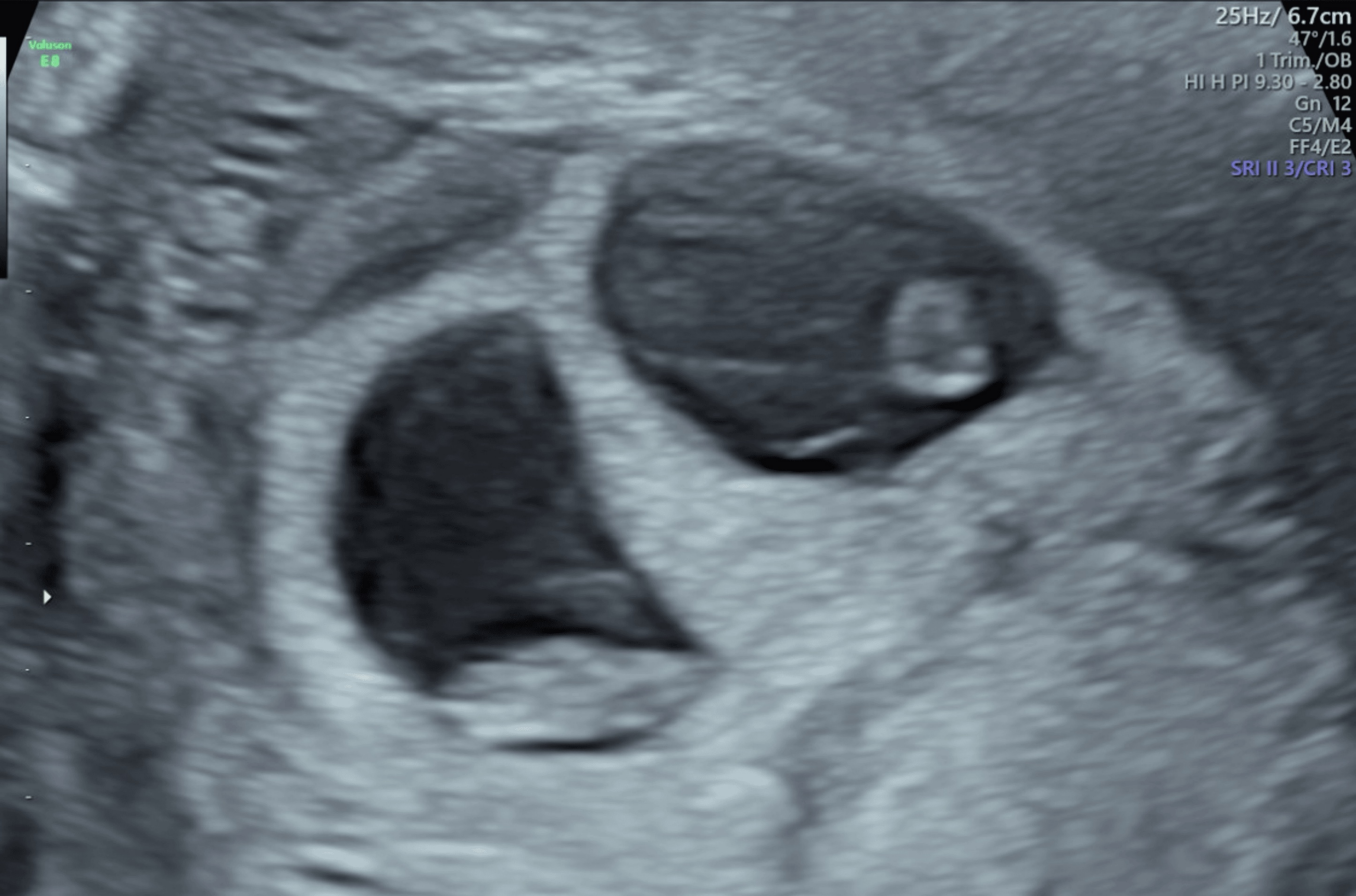
Pelvic ultrasound showing viable twin pregnancy

At seven weeks’ gestation, she experienced vaginal spotting; a pelvic ultrasound confirmed viable twin embryos. During routine antenatal follow-up, one foetus was noted to be developing normally (Figure 1), whilst the other showed an increased risk of trisomy; however, the patient declined invasive testing after counselling due to concerns about procedure-related risks.

At 23 weeks’ gestation, she presented with dyspnoea and palpitations with hypoxia. Laboratory investigations showed an elevated D-dimer. CTPA excluded pulmonary embolism but showed bilateral pulmonary infiltrates consistent with pneumonia. No organism was isolated, and the diagnosis was made radiologically. The scan was performed with appropriate precautions to limit foetal radiation exposure, including abdominal shielding and the use of a low-dose protocol tailored for pregnant patients. Following a week of intravenous antibiotic therapy, her pregnancy progressed without complications until late gestation.

At 36 weeks’ gestation (one day prior to a planned caesarean section), an assessment by her community midwife highlighted persistent maternal tachycardia (resting heart rate 112 bpm), and she was referred to the obstetric team. Upon arrival at the hospital, she developed sudden respiratory distress with hypoxia and pink frothy sputum. This was followed by cardiotocography (CTG) showing persistent late decelerations and reduced variability, indicative of foetal distress, requiring an emergency caesarean section under general anaesthesia. Two live neonates were delivered. One neonate was healthy; however, the second one was diagnosed with tracheoesophageal fistula, requiring transfer to a specialist paediatric centre where surgical correction was performed. Postoperative recovery was uneventful, and the neonate was discharged home within two weeks.

Approximately 20 minutes post-surgery (whilst in the theatre), she suffered massive PPH (estimated blood loss of 2.6 L), and a vaginal Bakri balloon was inserted to achieve haemostasis. She was transferred to the intensive care unit (ICU), where her condition deteriorated with increasing oxygen requirements.

Arterial blood gas showed mixed respiratory and metabolic acidosis (Table 1). Laboratory investigations showed leucocytosis, anaemia, thrombocytopenia, and coagulopathy with low fibrinogen levels and raised activated partial thromboplastin time (APTT) consistent with DIC (Table 2).

**Table 1:** Arterial blood gas results from admission to ICU until discharge pH: potential of hydrogen; PaO₂: partial pressure of oxygen; PaCO₂: partial pressure of carbon dioxide; ICU: intensive care unit

Blood gases	Post ICU admission	Post-extubation	Before discharge	Normal range
pH	7.27	7.35	7.46	7.35-7.45
PaO_2_	33.9 kPa	10.3 kPa	11.6 kPa	11.4-14.4 kPa
PaCO_2_	6.28 kPa	5.9 kPa	4.96 kPa	4.5-6.5 kPa
Lactate	3.3 mmol/L	1.3 mmol/L	0.6 mmol/L	0.5-1.6 mmol/L
Bicarbonate	20 mmol/L	24 mmol/L	26 mmol/L	21-28 mmol/L
Base excess	-5.1 mmol/L	-0.9 mmol/L	1.8 mmol/L	-2.0-2.0 mmol/L
Haemoglobin	92 g/L	112 g/L	109 g/L	120-160 g/L

**Table 2:** Blood results from admission to ICU until discharge WBC: white blood cell; HB: haemoglobin; APTT: activated partial thromboplastin time; INR: international normalised ratio; CRP: C-reactive protein; ICU: intensive care unit

	Post ICU admission	Post-extubation	Before discharge	Normal range
WBC	19.8 x 10⁹/L	11.6 x 10⁹/L	10.4 x 10⁹/L	3.5-10.0 x 10⁹/L
Neutrophils	17.3 x 10⁹/L	8.6 x 10⁹/L	7.4 x 10⁹/L	1.7-7.5 x 10^9^/L
HB	91 g/L	110 g/L	113 g/L	115-145 g/L
Platelets	139 x 10⁹/L	341 x 10⁹/L	408 x 10⁹/L	150-400 x 10⁹/L
APTT	1.3 ratio	1 ratio	1 ratio	0.8-1.2 ratio
INR	1 ratio	0.9 ratio	0.9 ratio	0.8-1.2 ratio
Fibrinogen	1.76 g/L	2.7 g/L	-	1.9-4.5 g/L
CRP	20.1 mg/L	6.8 mg/L	4.7 mg/L	<5.0 mg/L

**Figure 2:**
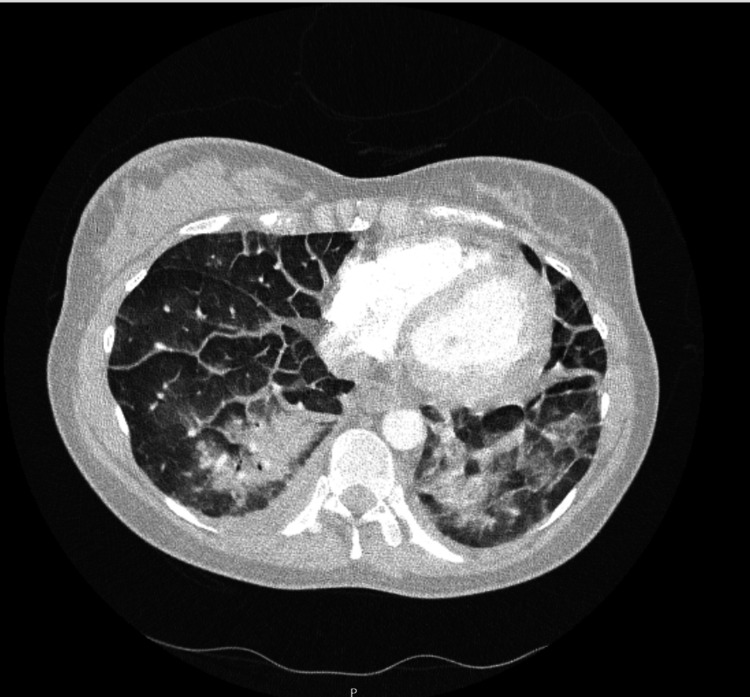
CTPA showing bilateral ground-glass opacities CTPA: computed tomography pulmonary angiogram

Repeat CTPA excluded pulmonary embolism but revealed bilateral, symmetrical ground-glass opacities in the perihilar and basal lung fields, coalescing into areas of lower lobe consolidation, with smooth interlobular septal thickening and small bilateral pleural effusions (Figure 2). Trace pericardial effusion was noted with no airway obstruction.

Transthoracic echocardiography in the ICU showed LV dysfunction with an EF of 40%-45%, mild LV and left atrial dilatation, and moderate to severe MR (Video 1).

**Video 1:** Transthoracic echocardiography showing acute mitral regurgitation

In the ICU, she was intubated and sedated with mechanical ventilation and required high oxygen support (FiO₂ 60%), positive end-expiratory pressure (PEEP) of 10 cm H₂O, and peak inspiratory pressure of 16 cm H₂O. Arterial blood gases revealed PaO₂ 10 and SO_2_ 96%. The patient was haemodynamically unstable, with a mean arterial pressure (MAP) of 48 mmHg requiring vasopressor support with high-dose norepinephrine (up to 0.4 mcg/kg/min) to maintain MAP around 60-65 mmHg, received transfusions of packed red blood cells and fresh frozen plasma, and was commenced on diuretic therapy to treat pulmonary oedema. After two days, the intravaginal balloon was removed as the vaginal bleeding had stopped. The mixed respiratory and metabolic acidosis has been improved. Antibiotics were continued.

On day two, we attempted to hold sedation, but she developed respiratory distress, tachypnoea, and tachycardia, with increasing oxygen requirements. Therefore, sedation was restarted, diuresis was continued to manage fluid overload, and nebuliser therapy was added. Her respiratory status gradually improved, norepinephrine was weaned, and her oxygenation gradually improved over the next 48 hours, allowing a reduction in FiO₂ to 21% and eventual extubation on day three. Urine output was maintained, and no renal support was required.

PPCM was considered, but several factors made it less likely: the cardiac dysfunction occurred acutely postpartum, resolved rapidly within days, and was accompanied by DIC and massive haemorrhage features uncharacteristic of PPCM. Moreover, imaging findings were more consistent with inflammatory lung injury than isolated cardiogenic pulmonary oedema.

Post-extubation arterial blood gas result parameters improved (Table 1). There was an improvement in the inflammatory markers, normalisation of white cell count, and coagulation (Table 2).

Cardiology reviewed her on multiple occasions. Repeat echocardiography before discharge showed persistent mild LV systolic impairment (EF 45%), hypokinesia of the septum and inferior wall, moderate MR, and a dilated right ventricle (RV) with preserved longitudinal function. She was discharged with heart failure medications, venous thromboembolism prophylaxis and antibiotics with cardiology, and obstetric outpatient follow-up.

A transoesophageal echocardiogram was performed two weeks post-discharge to better characterise MR and reassess LV function before stopping heart failure therapy, which showed preserved LV function with mild MR (effective regurgitant orifice 0.2 cm², regurgitant volume 20 mL, and vena contracta 0.27 cm) (Video 2). At the obstetric follow-up, a transvaginal ultrasound revealed an intraendometrial clot (21 × 10 × 17 mm) with no other abnormalities.

**Video 2:** Post-discharge transoesophageal echocardiography video showing normal mitral valve

All medications were stopped one month post-discharge, as she was asymptomatic, and the transoesophageal echocardiogram returned to normal.

## Discussion

This case illustrates the sudden, life-threatening nature of AFE in the peripartum period. Our patient experienced acute respiratory distress, rising oxygen demand, and atonic PPH shortly after an emergency caesarean section, raising suspicion for AFE [10].

AFE is a rare but fatal obstetric emergency (1-2 per 100,000 deliveries) and a leading cause of maternal mortality in high-resource settings [11]. Diagnosis is challenging due to overlapping symptoms-sudden respiratory failure, haemodynamic collapse, and DIC-with other postpartum emergencies [12].

Differential diagnoses included pulmonary embolism, PPCM, and primary haemorrhagic shock. Each can cause hypoxemia and cardiovascular collapse, but none fully accounts for the AFE triad: respiratory distress, severe hypotension, and coagulopathy with haemorrhage (Table 3).

**Table 3:** Comparison between AFE, PPCM, and PE CTPA: computed tomography pulmonary angiography; DIC: disseminated intravascular coagulation; LV: left ventricular; RV: right ventricular; MR: mitral regurgitation; AFE: amniotic fluid embolism; PPCM: peripartum cardiomyopathy; PE: pulmonary embolism; EF: ejection fraction; BNP: B-type natriuretic peptide

Feature	AFE	PPCM	PE
Onset	Sudden, peripartum (often during labour or shortly after delivery)	Subacute, usually in late pregnancy or early postpartum	Sudden onset, can occur antenatally or postnatally
Respiratory symptoms	Acute hypoxia, pink frothy sputum	Progressive dyspnoea, orthopnoea	Sudden dyspnoea, pleuritic chest pain
Haemodynamic status	Hypotension, shock, often with DIC	Variable, may present with heart failure	May cause hypotension or right heart strain in large PE
Coagulopathy	Common (DIC, low fibrinogen, thrombocytopenia)	Rare	Possible with massive PE but not typical
Imaging (CTPA)	Bilateral ground-glass opacities, no PE	Pulmonary oedema pattern if severe	Filling defect in pulmonary arteries
Echocardiography	Transient LV dysfunction, no RV strain, MR possible	Global LV dysfunction, EF < 45%, gradual recovery	RV dilation, hypokinesia, septal flattening
Cardiac biomarkers	Often normal or mildly elevated	Elevated BNP, troponin	Possible troponin rise in large PE
Response to treatment	Biphasic course, rapid deterioration, then recovery	Gradual improvement over weeks	Improves with anticoagulation and supportive care

Although initial leucocytosis was present (white blood cell (WBC) 19.8 x 10⁹/L), serial inflammatory markers supported non-infective causes. C-reactive protein (CRP) decreased from 20.1 to 4.7 mg/L within 72 hours. Procalcitonin was measured at 0.09 ng/mL (normal <0.1), further supporting the absence of systemic infection.

Diagnosing AFE in real time is a diagnosis of exclusion, as no definitive test or early marker exists [13]. Emerging biomarkers are being studied. Elevated serum tryptase and complement components (e.g., C3a and C5a) may support the diagnosis, reflecting mast cell degranulation and complement activation. However, these are not routinely available and were not measured in this case. In fatal cases, histopathological examination showing foetal squamous cells or lanugo in maternal pulmonary vasculature may confirm the diagnosis post mortem, but such findings can be non-specific [12]. Clinicians must rule out other causes (e.g., negative bedside echocardiography/CT for pulmonary thrombus and no uterine rupture or anaesthetic complications) whilst recognising the characteristic pattern: sudden hypoxia, hypotension, altered mental status (often with seizures or loss of consciousness), and rapid-onset coagulopathy during labour or shortly postpartum [14].

In our case, the timing, simultaneous cardiovascular collapse, and coagulopathy were classic for AFE. Massive haemorrhage from uterine atony and DIC, commonly seen in AFE, was also present. Registry data show that maternal dyspnoea and massive obstetric haemorrhage are the most frequent presenting signs [15]. This overlap can make AFE initially appear as isolated PPH, delaying recognition. The presumptive diagnosis of AFE was made here after excluding pulmonary embolism and identifying no other cause for the combined respiratory and haemorrhagic collapse.

AFE as an immune-mediated 'anaphylactoid' syndrome

Originally mischaracterised as an ‘embolism’, AFE is now understood as a rare, immune-mediated, anaphylactoid syndrome triggered by exposure to foetal antigens. The earlier belief that physical obstruction of the pulmonary vasculature by amniotic debris was responsible has been challenged by autopsy studies showing foetal cells in maternal lungs even in asymptomatic women [12]. Consequently, AFE has been reclassified as a non-thrombotic, immune-related condition [13,14].

Current evidence supports biphasic pathophysiology: an initial phase marked by acute pulmonary hypertension and RV failure from vasoactive mediator release, followed by myocardial depression, systemic vasodilation, and consumptive coagulopathy culminating in left-sided heart failure and haemorrhagic DIC [13]. Echocardiographic findings may include acute RV dysfunction in the absence of pulmonary embolism and rapid LV recovery, both of which can support the diagnosis of AFE and help exclude other causes.

The immunologic hypothesis posits that amniotic components trigger a systemic inflammatory cascade, leading to cytokine release, cardiovascular collapse, and activation of the coagulation pathway. Whilst this theory informs current management strategies resembling anaphylaxis treatment (e.g., epinephrine, steroids, and supportive care), it remains theoretical and lacks validation from large-scale epidemiologic studies. Notably, syncytial knots-though more common in AFE cases-are non-specific histological findings and are not currently considered markers of AFE risk.

Some studies suggest a potential link between ART and AFE, particularly in donor oocyte pregnancies. However, this association may be confounded by coexisting factors such as advanced maternal age and multiple gestations. Case reports often do not account for these variables, limiting causal inference. It has been proposed that antigen exposure may occur through uteroplacental breaches during procedures like caesarean section or manual placental removal, which could allow foetal material to enter the maternal circulation suddenly. Whilst registry data have reported increased AFE incidence in ART pregnancies, these findings must be interpreted cautiously [15,16]. Many cases lack confirmation of donor oocyte use, and details such as sample size and collection period are often unspecified.

Recurrent AFE is exceedingly rare, likely because each pregnancy presents a distinct foetal antigenic profile, meaning previous survival does not necessarily increase recurrence risk.

Imaging considerations in AFE

Whilst no imaging can definitively diagnose AFE, certain findings can support the diagnosis or exclude other causes. CTPA, often performed to rule out pulmonary embolism in postpartum collapse, may reveal diffuse bilateral ground-glass opacities rather than focal thromboembolism [10].

In our case, CTPA showed bilateral pulmonary infiltrates without thrombus-an indication of AFE. The first published CT images of acute AFE in 2010 displayed homogeneous ground-glass opacification in both lungs, primarily peripheral, resembling acute respiratory distress syndrome (ARDS). These infiltrates reflect acute lung injury and permeability oedema from the immunologic response [11].

Donor oocyte pregnancy and maternal immune activation

Pregnancies conceived with donor oocytes have a distinct immunologic profile, with higher rates of obstetric complications compared to naturally conceived or autologous IVF pregnancies. In donor oocyte pregnancies, the foetus is genetically unrelated to the mother, functioning as a complete allograft from the maternal immune system’s perspective [12,15,16].

In spontaneous conception or standard IVF (using the woman’s own eggs), half of the foetal antigens are maternal, promoting immune tolerance. However, donor egg embryos introduce entirely ‘foreign’ antigens (from the donor’s genome and the partner’s sperm), intensifying maternal-foetal immune interactions.

Women with donor egg pregnancies present a unique immunological environment. The complete lack of maternal HLA similarity in such pregnancies leads to heightened maternal immune activation, with increased levels of inflammatory cytokines (e.g., TNF-α, IL-6, and IFN-γ) and placental histological features such as chronic villitis and syncytial knots. These elements may increase the risk of trophoblastic and amniotic debris entering maternal circulation and triggering a maladaptive immune response. This immune hypersensitivity is believed to play a central role in the pathogenesis of AFE, reframing it as an anaphylactoid reaction rather than a mechanical embolism. Donor egg placentas also show a high density of syncytial knots-clusters of apoptotic syncytiotrophoblasts released into the maternal circulation, which may indicate placental stress [15].

This heightened immune activation may increase trophoblastic debris in maternal circulation, potentially triggering systemic inflammation or coagulopathy, which could contribute to complications like preeclampsia and AFE.

Donor oocyte pregnancies are consistently associated with higher risks of first-trimester bleeding, placental abnormalities, gestational diabetes, caesarean delivery, and PPH. A cohort study reported higher rates of atony and haemorrhagic complications in donor-conceived pregnancies, likely due to placental dysfunction (e.g., placenta accreta or atony) [17]. These findings highlight donor oocyte gestation as a state of heightened maternal-foetal immunologic tension with significant clinical implications.

Donor oocyte IVF: a potential risk factor for AFE?

Given the immune-mediated nature of AFE and the heightened immune activation in donor oocyte pregnancies, it has been hypothesised that donor IVF may increase the risk of AFE. The theory suggests that greater maternal-foetal antigen mismatch in donor egg pregnancies may elevate the risk of foetal material entering maternal circulation, triggering a catastrophic immune response characteristic of AFE.

Donor egg recipients are often older and have higher rates of obstetric complications-both established risk factors for AFE, including advanced maternal age and placental pathologies [2]. However, beyond these factors, maternal-foetal immunologic incompatibility in donor IVF could independently contribute to AFE. Any breach in the uteroplacental interface (e.g., during placental separation or invasive procedures) might expose the maternal immune system to a high burden of foreign foetal antigens, including donor-derived cells, trophoblastic fragments, and amniotic fluid components. This could trigger an exaggerated immune response, including complement activation and cytokine release.

Case reports support this hypothesis. Schutte et al. described a fatal AFE in a 44-year-old woman undergoing donor oocyte IVF [16]. A nationwide review of Dutch data (1984-2008) revealed that 24% of maternal deaths in IVF pregnancies involved donor gametes [18]. Similarly, a Danish report documented a suspected AFE with DIC during a caesarean delivery in a donor oocyte pregnancy, highlighting the need for awareness of AFE in ART pregnancies [19].

Large registry studies have not conclusively identified donor IVF as a significant risk factor for AFE, likely due to the small proportion of donor-conceived pregnancies among all births and AFE’s rarity. However, ART in general is noted as a potential risk factor, with the Amniotic Fluid Embolism Foundation listing ‘assisted fertility (IVF, intrauterine insemination (IUI), and egg and/or sperm donation)’ among AFE-associated conditions [20].

The challenge remains in distinguishing the immunologic risks of donor egg IVF from confounding factors such as advanced maternal age, multiple gestation, and placental abnormalities. Further research is needed to clarify the specific role of donor IVF in AFE risk.

## Conclusions

AFE is a rare but life-threatening obstetric emergency with significant implications for both maternal and foetal outcomes. This case of suspected AFE in a donor oocyte IVF pregnancy underscores the intersection of advanced reproductive technology and critical care. Although AFE is unpredictable, its severity warrants vigilance and knowledge sharing in each case. Clinicians should maintain a high index of suspicion in patients with known risk factors, such as advanced maternal age, multiparity, multiple gestation, and ART, particularly donor oocyte IVF, when presenting with acute respiratory distress, hypotension, and coagulopathy with haemorrhage. Early recognition, rapid resuscitation, expedited delivery when indicated, and ICU monitoring are critical for improving outcomes. Continued case reporting and collaborative research are essential to enhance understanding of AFE, develop standardised diagnostic criteria, and explore potential preventive strategies, such as immunologic markers, placental pathology, and ART-specific surveillance. Until definitive prevention or diagnostic tools are available, interdisciplinary preparedness and supportive care remain central to optimising patient safety.
